# Effects of combination therapy of antithrombin and thrombomodulin for sepsis-associated disseminated intravascular coagulation: a systematic review and meta-analysis

**DOI:** 10.1186/s12959-023-00579-z

**Published:** 2024-01-15

**Authors:** Takaaki Totoki, Yuto Makino, Kazuma Yamakawa, Hiroyuki Koami, Takeshi Wada, Takashi Ito, Toshiaki Iba

**Affiliations:** 1 Department of Anesthesiology & Critical Care Medicine, Kyushu University, Fukuoka, Japan; 2 Department of Preventive Services, Kyoto University Graduate School of Medicine, Kyoto, Japan; 3 Department of Emergency and Critical Care Medicine, Osaka Medical and Pharmaceutical University, Osaka, Japan; 4 Advanced Emergency Care Center, Saga University Hospital, Saga, Japan; 5 Division of Acute and Critical Care Medicine, Department of Anesthesiology and Critical Care Medicine, Hokkaido University Faculty of Medicine, Sapporo, Japan; 6 Department of Biomedical Laboratory Sciences, Faculty of Life Sciences, Kumamoto University, Kumamoto, Japan; 7 Department of Emergency and Disaster Medicine, Juntendo University Graduate School of Medicine, Tokyo, Japan

**Keywords:** Sepsis, Disseminated intravascular coagulation, Antithrombin, Thrombomodulin

## Abstract

**Background:**

Disseminated intravascular coagulation (DIC) syndrome is a highly lethal condition characterized by the complication of multiple organ damage. Although the effects of combined antithrombin (AT) and recombinant thrombomodulin (rTM) on DIC syndrome have previously been examined, the results are inconsistent and inconclusive. Therefore, we conducted a systematic review on the combined administration of AT and rTM for the treatment of septic DIC to investigate the superiority of the combination therapy over either AT or rTM monotherapy using a random-effects analysis model.

**Method:**

We searched electronic databases, including Medline, Cochrane Central Register of Controlled Trials, Scopus, and Igaku-Chuo Zasshi (ICHU-SHI) Japanese Central Review of Medicine Web from inception to January 2022. Studies assessing the efficacy of combined AT and rTM were included. The primary outcome was all-cause mortality, and the secondary outcome was occurrence of serious bleeding complications compared to monotherapy. We presented the pooled odds ratio (OR) or hazard ratio (HR) with 95% confidence intervals (CI) depending on reporting results in each primary study.

**Results:**

We analyzed seven enrolled clinical trials, all of which were observational studies. Combination therapy had a non-significant favorable association with lower 28-day mortality compared to monotherapy (HR 0.67 [0.43–1.05], OR 0.73 [0.45–1.18]). The *I*^*2*^ values were 60% and 72%, respectively, suggesting high heterogeneity.

As a secondary outcome, bleeding complications were similar between the two groups (pooled OR 1.11 [0.55–2.23], *I*^*2*^ value 55%).

**Conclusions:**

Although the findings in this analysis could not confirm a statistically significant effect of AT and rTM combination therapy for septic DIC, it showed a promising effect in terms of improving mortality. The incidence of bleeding was low and clinically feasible. Further research is warranted to draw more conclusive results.

**Trial registration:**

This study was registered in the University Hospital Medical Information Network (UMIN) Clinical Trials Registry (UMIN ID: 000049820).

**Supplementary Information:**

The online version contains supplementary material available at 10.1186/s12959-023-00579-z.

## Background

Disseminated intravascular coagulation (DIC) is a frequent and highly lethal complication, affecting 51% of sepsis cases treated in the intensive care unit (ICU). Since DIC is not merely a complication but is involved in the pathogenesis of organ dysfunction, mortality increases approximately two-fold when it accompanies sepsis [1].

Treatment strategies for septic DIC vary widely among guidelines from different countries [2, 3]. While some guidelines recommend only supportive therapies, the Japanese sepsis guidelines recommend early detection and early initiation of anticoagulation by antithrombin (AT) or recombinant human soluble thrombomodulin (rTM) [3].

AT in plasma inhibits thrombin and several other serine protease coagulate factors, and it exerts antiplatelet effects via stimulating prostacyclin production by using vascular endothelial cells [4, 5]. Further, AT administration has been suggested to protect vascular endothelial cells [6], thereby potentially improving the prognosis [7].

Activated protein C (APC)/TM system is another important physiological anticoagulant system. Activated protein C (APC) exerts an anticoagulant effect by degrading active factor V and active factor VIII using protein S as a cofactor [8]. TM is a glycoprotein and is present in vascular endothelial cells; its expression is known to be reduced considerably in sepsis. Since protein C is activated by the binding of thrombin and TM, reduced thrombomodulin leads to procoagulant changes in sepsis. Thus, external administration of rTM may facilitate DIC withdrawal and reduce mortality in patients with septic DIC [9, 10].

Basic experiments suggested that the combination of AT and rTM might improve prognosis compared to a single administration of either drug [11, 12], showing that the produced thrombin might be sufficient to activate protein C. Although the effects of combination therapy have been examined in clinical studies, previous studies have demonstrated mixed results [13–16], and the synergistic or additive effects of two anticoagulants remain to be clarified. Therefore, we conducted a systematic review on the combined administration of AT and rTM for the treatment of septic DIC to evaluate the usefulness of this combination therapy.

## Materials and methods

### Protocol and registration

This study was registered in the University Hospital Medical Information Network (UMIN) Clinical Trials Registry, which is the largest clinical trial registry in Japan (UMIN ID: 000049820). Ethical approval and consent to participate were not required for this systematic review.

### Search strategy

Databases, including MEDLINE (PubMed, 1966–January 2023), Cochrane Central Register of Controlled Trials (through January 2023), Science Citation Index Expanded (1900–January 2023), and Igaku-Chuo Zasshi (ICHU-SHI) Japanese Central Review of Medicine Web (1983–January 2023) were searched. Since the drugs are only approved in Japan, non-English articles, such as those in Japanese, were included in this analysis.

Each search query included the following terms: “thrombomodulin,” “Recomodulin” (brand name of rTM), “ART-123” (code name of rTM), “sepsis,” “systemic inflammatory response syndrome,” and “disseminated intravascular coagulation;” these terms were also searched in Japanese characters in the ICHUSHI database. Additional file 1 shows the specific details regarding the search strategies and results.

We also manually searched the references of the articles of interest to identify other potentially relevant studies. This study was conducted in accordance with the Preferred Reporting Items for Systematic Reviews and Meta-Analyses [17].

### Study selection and inclusion criteria

Two independent reviewers (T.T. and Y.M.) screened the abstracts and titles of the studies and subsequently reviewed the full-text articles for inclusion. Studies with the following characteristics were included:

1. Study types: randomized controlled trials (RCT) or observational studies, which are prospective/retrospective cohort studies with concurrent controls or cohort studies with historical controls.
2. Population: Patients with septic DIC. Results for RCTs that included sepsis in general or mixed DIC due to other underlying diseases, such as trauma, leukemia, and so on, were considered only if the results of the subgroup analysis of “septic DIC” were presented in the main or separate paper.
3. Intervention: Combination AT and rTM therapy.
4. Control: Treatment with rTM or AT administration.

### Risk of bias in individual studies

Three independent reviewers (T.T., Y.M., and H.K.) assessed the risk of bias (RoB) in individual studies to determine the methodological quality of the articles, and disagreements were resolved through discussion and consensus. Uniform criteria were applied to evaluate the RoB associated with the Cochrane Collaboration “risk of bias” tool. Because all the included studies were observational studies in this analysis, we used the ROBINS-I tool, which has been validated in nonrandomized studies, to assess the RoB of the studies included in this meta-analysis [18]. Studies were assessed as “low,” “moderate,” “high,” “serious,” or “critical” risk for each domain. Importantly, ROBINS-I bias assessments were made by comparing a given study and a theoretical RCT with an ideal design for the study question—the latter representing the standard as a “low-risk” study. For this reason, the “low-risk standard” for bias assessment was defined as an ideal observational study.

### Data extraction

Two independent reviewers (T.T. and Y.M.) extracted the data using a standardized data extraction sheet, and disagreements were resolved by discussion and consensus. We identified the primary author’s name, year of publication, inclusion and exclusion criteria, patient population, as well as the use of AT and rTM. The primary outcome measure was all-cause mortality at 28 or 30 days after study entry or in-hospital mortality. The secondary outcome measure was serious bleeding complications, defined as fatal or life-threatening complications as proposed by the authors of the individual studies, and recovery from DIC. The definition of DIC followed that by the authors of the primary study, such as the Japanese Association for Acute Medicine DIC diagnostic criteria. Recovery from DIC was defined as a negative result for each DIC diagnostic criterion on day 7.

### Statistical analysis and data synthesis

We presented the results of all analyses according to a random-effects model because this model incorporates statistical heterogeneity. The random-effects model provided a more conservative estimate of the pooled effect size than a fixed-effects model. For dichotomous variables (e.g., mortality, serious bleeding complications, and DIC resolution rate), the odds ratio (OR) or hazard ratio (HR) were expressed as point estimates with 95% confidence intervals (CI) and P-value depending on reporting results in each study. All OR and HR referred to the risk of the combination group compared with the control groups. For observational studies, only those that presented results adjusted for confounding factors were included in the meta-analysis.

All statistical analyses, including the RoB within studies and/or across studies, were performed using Review Manager Version 5.4. (RevMan; The Cochrane Collaboration 2012, The Nordic Cochrane Centre, Copenhagen, Denmark). The level of statistical significance was set at a *P*-value < 0.05.

## Results

### Literature search

**Fig. 1:**
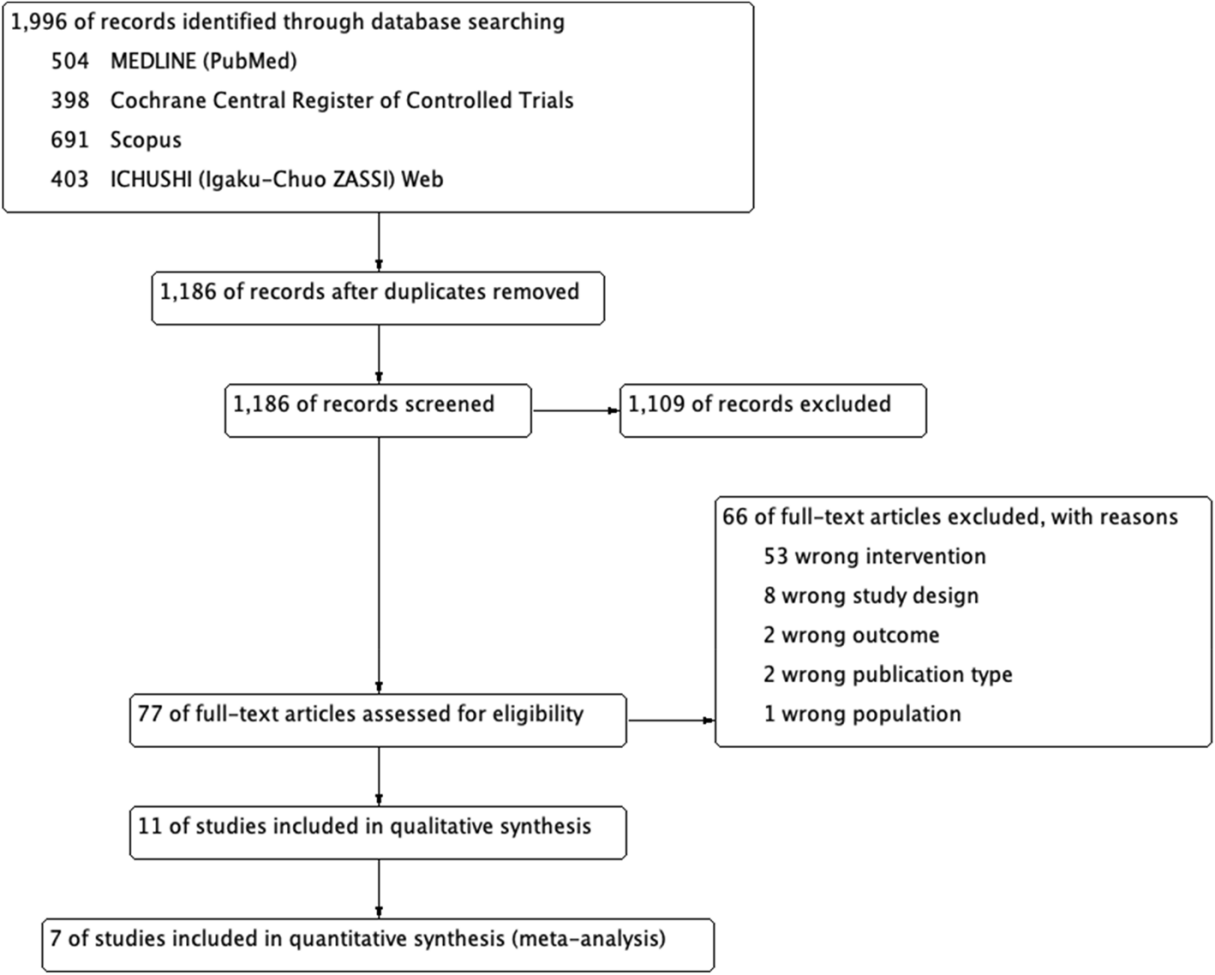
Preferred reporting items for systematic reviews and meta-analyses flow chart

Figure 1 shows the flow of the PRISMA flowchart selection. The initial search produced 1996 articles. After excluding duplicates, we identified 1186 studies from electronic databases, among which 77 were retained based on the assessment of the study titles and abstracts. According to the review of full-text articles, 66 studies were excluded because they did not meet the inclusion criteria (i.e., the patient did not have sepsis, not using a target drug, study conducted with a different study design, or wrong outcome inappropriately). Finally, 11 studies were included in the qualitative synthesis [13–16, 19–25]. Amongst these, four reported only unadjusted results [19, 21, 22, 24]; hence, seven studies were included in the quantitative synthesis (Fig. 1).

All of the included studies were observational and retrospective in nature. Four studies were written in Japanese, and the rest in English. Seven studies had AT alone as a control group, three studies had rTM alone as a control group, and one study had both AT alone and rTM alone as control groups. More details about the characteristics of the included studies are provided in Table 1.

**Table 1:** Main characteristics of all studies included in the meta-analysis

**Author **	**Year**	**Design**	**Number of sites**	**Country**	**Sample Size**	Age*	**Types of intervention**	**Types of control**	**Mortality**	**Bleeding complications**
Sakurai	2013	Retro	1	Japan	60	NI	rTM: 380 U/kg/day, AT: 1500U/day	rTM alone	C:15% (3/20)	N/A
									M:25% (10/40)	
Sawano	2013	Retro	1	Japan	111	C: 69.1(±2.2), M: 68.1 (±2.2)	rTM:130- 380 U/kg/day, AT: 1500U/day	AT alone	C:14% (7/50)	N/A N/A
									M:40% (24/60)	
Takehara	2014	Retro	1	Japan	13	C: 81, M: 62	rTM:380U/kg/day, AT: 1500U/day	AT alone	C:22.2% (8/36)	N/A
									M:20% (5/25)	
Hosomi	2014	Retro	1	Japan	20	C: 73.5(55-81.5), M: 70(60.75-73)	rTM:130- 380 U/kg/day, AT: 1500U/day	AT alone	C:0% (0/10)	N/A N/A
									M:0% (0/10)	
Iba	2016a	Retro	Multi, not specified	Japan	1026	NI	rTM:130-380 U/kg, AT: 1500 or 3000U/day	AT alone	C:22.4% (51/228)	OR: 0.391
									M:28.7% (81/282)	CI: 0.092-1.6617
Iba	2016b	Retro	Multi, not specified	Japan	159	NI	rTM:130-380 U/kg, AT: 1500 or 3000U/day	AT alone	N/A	C: 4.11% (3/73)
										M: 4.17% (2/48)
Iba	2017	Retro	Multi, not specified	Japan	510	C:72.3(±15.6)M:71.7(±14.7)	Not specified	AT alone	HR: 0.55	N/A
									CI: 034-0.8897	
Morita	2019	Retro	1	Japan	56	C:77.6(±10.2)M: 83.0 (±8.5)	rTM:130-380 U/kg, AT: 500U/kg/day	rTM alone	C:5% (2/40)	N/A
									M:0% (0/16)	
Suzuki	2020	Retro	462	Japan	662,387	C:71.2(±14.0)M:72.0(±14.2)	Not specified	rTM alone	C:40.2% (76/189)	N/A
									M:45.5% (86/189)	
Umegaki	2020	Retro	More than 1000	Japan	2222	C:70.3(±13.4)M:68.9(±14.1)	Not specified	AT alone	OR: 1.0655	N/A
									CI: 0.9012-1.2597	
Umemura	2019	Retro	42	Japan	1432	C: 72 (62-80), M (AT): 72 (63-80), M (rTM): 72 (62-78)	Not specified	AT alone or rTM alone	AT	AT
									HR: 0.9708	OR: 1.0102
									CI: 0.6024-1.5646	CI: 0.5619-1.8161
									rTM	rTM
									HR: 0.9174	OR: 1.923
									CI: 0.5494-1.5319	CI: 0.9709-3.8089

*Pro *prospective study, *Retro *retrospective study, *NI *Not information, *I *Intervention group, *C *Control group, *AT *Antithrombin, *rTM *Recombinant human soluble thrombomodulin, *N/A* Not available

^*^Presented as mean ±standard deviation or median (Interquartile range)

### Risk of bias within studies

The consensus ROBINS-I assessments of all 11 included studies are summarized in Table 2. Of these, four studies had a critical RoB, four studies had a serious RoB, and three studies had a moderate RoB. No studies had a low RoB. Notable bias was identified in the “bias due to confoundings” domain, mainly because of the nonrandomized nature of studies and a lack of sufficient confounding adjustment. Moreover, in the “bias due to missing” domain, most studies were considered to have a high RoB or no information as only complete case analyses were performed and missing data were not reported. Classification of intervention and outcome measurement domains demonstrated less RoB.

**Table 2:** Risk-of-bias assessment in 11 studies using ROBINS-I

**Author **	**Bias due to confounding**	**Bias in selection of participants into the study**	**Bias in classification of interventions**	**Bias due to deviations from intended interventions**	**Bias due to missing data**	**Bias in measurement of outcomes**	**Bias in selection of the reported result**	**Overall**
Sakurai 2013	Critical	Moderate	Low	Moderate	Serious	Low	Moderate	Critical
Sawano 2013	Serious	Moderate	Low	Moderate	Serious	Low	Moderate	Serious
Takehara 2014	Critical	Moderate	Low	Moderate	NI	Low	Moderate	Critical
Hosomi 2014	Critical	Moderate	Low	Moderate	Critical	Low	Moderate	Critical
Iba 2017	Moderate	Moderate	Low	Moderate	Serious	Low	Moderate	Serious
Umemura 2018	Moderate	Moderate	Low	Moderate	NI	Low	Moderate	Moderate
Morita 2019	Critical	Moderate	Low	Moderate	NI	Low	Moderate	Critical
Suzuki 2020	Moderate	Moderate	Low	Moderate	NI	Low	Moderate	Moderate
Umegaki 2020	Moderate	Moderate	Low	Moderate	NI	Low	Moderate	Moderate
Iba 2016a	Moderate	Moderate	Low	Moderate	Serious	Low	Moderate	Serious
Iba 2016b	Serious	Moderate	Low	Moderate	Serious	Low	Moderate	Serious

### Mortality

Mortality was evaluated in ten studies. Of the ten studies, three studies were evaluated with hazard ratios (two 28-day mortality [13, 20], one in-hospital mortality [16]), while three studies were evaluated with adjusted odds ratios (one 28-day mortality [14], two in-hospital mortality [15, 25]), and four studies that only presented unadjusted odds ratios or the number of outcomes in the intervention and control groups. One study presenting HR reported the two comparison groups (AT alone and rTM alone) [16]; hence, both arms were included in the meta-analysis.

**Fig. 2:**
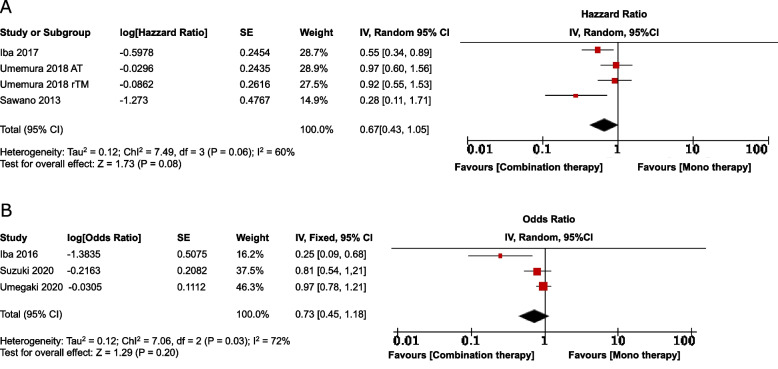
Forest plot of random-effect analysis comparing mortality rates for combination therapy and monotherapy for septic DIC. A Integrated hazard ratio results. B: Integrated odds ratio results. Disseminated intravascular coagulation: DIC

We calculated the pooled HR for studies reporting HR, which was 0.67 (95% CI of 0.43–1.05) (Fig. 2A), and the *I*^*2*^ value (60%) suggested substantial heterogeneity. We also calculated the pooled OR for studies reporting OR, which was 0.73 (95% CI 0.45–1.18) (Fig. 2B), and the *I*^*2*^ value (72%) suggested substantial heterogeneity. However, these results indicated a trend toward the usefulness of combination therapy. We could not perform predefined subgroup analyses due to inadequate data and the limited number of included studies. Additional File 1 presents the number of outcomes in the intervention and control groups for studies reporting only unadjusted results. Meanwhile, the analysis results for respective drugs are presented in Supplemental Fig. 1A, B, C, D. In addition, the results of Umemura et al. [16] comparing combination therapy with no anticoagulation therapy result are described in Supplemental Fig. 1E.

### Bleeding complications

**Fig. 3:**
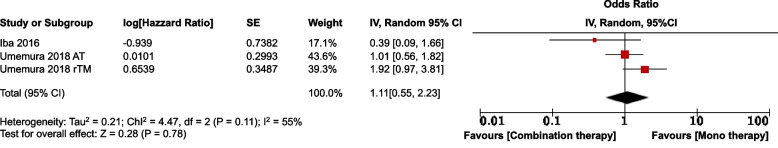
Forest plot of random-effects analysis comparing bleeding complications of combination therapy versus monotherapy for septic DIC. Disseminated intravascular coagulation: DIC

Bleeding complications were evaluated in two studies with adjusted ORs. One study reported the two comparison groups (AT alone and rTM alone) (18); hence, we included both arms in the meta-analysis. We calculated the pooled OR, which was 1.11 (95% CI 0.55–2.23) (Fig. 3), and the *I*^*2*^ value (55%) suggested moderate heterogeneity.

### Recovery from DIC

Recovery from DIC was evaluated in three studies. All three studies only presented unadjusted results, and we could not perform a meta-analysis. The number of outcomes in the intervention and control groups for each study are presented in Additional File 1.

## Discussion

### Principal findings

This study examined the usefulness of combination therapy with AT and rTM for septic DIC. The articles analyzed were all observational studies. Based on our study, combination therapy tended to improve mortality, although there was no statistical difference in mortality. There was also some concern that the combination of anticoagulant AT and rTM would increase bleeding complications. However, the results of this reviews suggest that bleeding complications do not increase with combined therapy. Heterogeneity amongst the included studies was also high.

### Mortality

This is the first systematic review and meta-analysis examining the efficacy and adverse events of AT + rTM for septic DIC. As no prior RCT has examined the effect of the combination therapy, the studies included were all observational studies.

In six studies that examined mortality, three presented results in terms of hazard ratios, and another three presented results in terms of adjusted odds ratios. In each of these studies, the combination of AT and rTM tended to reduce mortality compared with monotherapy, but the differences were not statistically significant.

Among the HRs examined, Sawano showed that combination therapy was particularly effective [20]. This single-center retrospective study included 111 patients (60 receiving monotherapy and 51 receiving combination therapy). One possible reason for the better results in combination therapy was the unevenness of patient distribution. The combination therapy group included more cases from 2009, whereas the monotherapy group (AT monotherapy) included more cases from 2006–2008, prior to the launch of rTM.

Iba et al. performed a similar study showing the effectiveness of combination therapy [13]. The study was a multicenter post-marketing study of AT consisting of 258 patients (129 monotherapy and 129 combination therapy).

These two studies showed significantly lower 28-day mortality in patients treated with combination therapy. Meanwhile, Umemura et al. [16] examined in-hospital mortality in a multicenter retrospective cohort study conducted in 42 ICUs in Japan with 808 patients and reported similar mortality in the combination therapy group and monotherapy group. However, both groups did not exhibit equal disease severity, and the combination therapy group was observed to have higher SOFA scores and DIC scores.

Among those studies examined with adjusted ORs, Iba’s study [14] analyzed 459 patients (monotherapy with AT 372 and combination therapy, 87) and found an improved prognosis with combination therapy. Suzuki [15] utilized the Diagnosis Procedure Combination database in Japan and constructed a matched pair of 378 patients with pneumonia-based septic DIC treated by anticoagulants (189 each, rTM monotherapy group and combination therapy group). In this study, although the difference was not statistically significant, the combination therapy group demonstrated lower mortality (40.2% vs. 45.5%).

Umegaki [25] utilized DPC data and examined the effect of combination therapy in 2222 patients (1017 in monotherapy with AT and 1205 in combination therapy). Again, the superiority of the combination therapy was not confirmed (OR: 0.97, 95% CI 0.78–1.21;* P* = 0.81). In this study, patients with septic DIC and with ventilator management were included, but the deviation of severe cases was not mentioned.

The present meta-analysis included highly heterogeneous studies (hazard ratio *I*^*2*^ = 60%; adjusted OR = 72%) with very different effect sizes across studies. Furthermore, we could not integrate all the studies due to the mixture of outcomes reported in HR and OR. However, both meta-analyses indicate that combination therapy tends to improve prognosis. Considering the mechanism of combination therapy, since antithrombin binds irreversibly to thrombin, it is suggested that AT administration may attenuate the APC-producing effect expected due to rTM by blocking the binding of thrombin to rTM. However, the clinical data in this study suggest that this view may not always be the case.

Since the studies included were all observational studies, there were some critical limitations. First, the treatment selection was unclear and was decided by the physicians in most of the studies. Although we were unable to confirm this, since severe cases were generally treated with combination therapy, it is unlikely that the combination therapy included more less-severe cases. Second, the treatment regimen was not consistent. The order of AT or rTM, whether given concomitantly or sequentially, and the time intervals between treatments were not clearly specified.

Owing to statistical issues, heterogeneous treatment regimens, and the lack of high quality, it is impossible to draw conclusions from the present study. However, we performed a systematic survey of the presently available data and observed that almost all the studies tended to show the beneficial effect of combination therapy. Therefore, we believe that combination therapy is potentially superior to monotherapy. Additionally, combination therapy has been indicated to be more effective in patients with severe thrombocytopenia and AT deficiency [26].

Wada et al. also reported that combination therapy may be useful for patients with low antithrombin and low fibrinogen [27]. High-quality observational studies and RCTs are necessary to make a recommendation in the future.

### Bleeding complications

In patients with sepsis-related coagulation disorders, the consumption of coagulation factors and platelets results in a bleeding tendency [23, 24]. Therefore, bleeding complications due to anticoagulation therapy are the main concern of clinicians. In the previous studies, the incidence of bleeding was sufficiently low, with AT and rTM used individually [14]. However, the risk of bleeding may increase when both anticoagulants are combined. In this study, the increase in bleeding complications was not observed in combination therapy. However, the effect sizes of the two studies differed significantly, and since the studies were moderately high heterogeneous (*I*^*2*^ = 55%), the quality of the evidence was low.

The three studies used in the analysis are two large Japanese studies and a multicenter post-marketing survey [14, 16]; we therefore consider our results to be reliable. Although regarding bleeding complications integrated results suggested that combination therapy is not inferior to monotherapy, this result should be interpreted with caution due to the moderately high heterogeneity among the studies.

### Recovery from DIC

With regard to DIC withdrawal rates, three studies were eligible. However, since they were not adjusted by confounders, the variability in the results was large, and there was a large amount of missing data; therefore, we thought that it might not be appropriate to perform a meta-analysis. The large number of missing data may be a result of the impossibility of collecting the necessary data to assess DIC withdrawal since all the studies included in this analysis were observational studies. However, the rate of recovery from DIC is an important clinical item, as it is one of the key indicators to assess the effectiveness of treatment and prognosis. Therefore, high-quality observational and prospective studies should be carried out in the future to examine the effect of combination therapy on DIC withdrawal.

#### Clinical application of the findings

Japanese Clinical Practice Guidelines for Management of Sepsis and Septic Shock recommend the administration of AT or rTM for DIC. In clinical practice in Japan, AT and rTM combination therapy has been used at some facilities, and there have been various reports on the usefulness of this therapy [13–16]. However, there is no consensus on the usefulness of combination therapy, and there are no guidelines discussing combination therapy, which is why this study was conducted.

The balance of the apparent benefits and harms of combination therapy for the treatment of septic DIC patients found in this study suggests that there is validity for its clinical use.

#### Limitations of the study

Our study characterized study bias using the ROBINS-I assessment since all studies on combination therapy were nonrandomized studies, allowing for a more nuanced understanding of the study findings and similar publications in the field. Moreover, all the studies included in this review were conducted in Japan with very high heterogeneity. Thus, the generalizability of our findings to other countries remains uncertain.

Moreover, while there may have been no published studies with negative results or ineffectiveness regarding the combination therapy of AT and rTM, the possibility of the existence of published papers showing its effectiveness cannot be ruled out. In addition, although the effects and adverse events of combination therapy should be examined and compared to those of patients treated without anticoagulant therapy, we were unable to find such a study. Therefore, we compared the efficacy of combined therapy to that of either AT or rTM monotherapy.

Besides the seven studies discussed in this report, two other studies compared combination therapy with monotherapy [28, 29]. However, since the results were shown only with Kaplan–Meier curves, OR and HR were not presented, and these studies were excluded from the analysis. Furthermore, although these studies reported favorable outcomes on combination therapy, their results might differ if additional data were available.

## Conclusions

Although the risk of bleeding did not increase, the present meta-analysis could not show statistically significant benefits of combination therapy with AT and rTM in patients with septic DIC in terms of mortality improvement. The fundamental limitation of this study is the lack of RCTs and high-quality observational studies. However, since almost all the studies tended to show a favorable trend, it seems reasonable to conclude that combination therapy of AT and rTM for the treatment of patients with septic DIC might be superior to monotherapy. Further studies are required to provide robust evidence.

### Supplementary Information

**Additional file 1: Supplementary Table 1.** Description of data: Specific details regarding the search strategies and results. **Supplementary Table 2.** Description of data: Number of outcomes comparing mortality rates for combination therapy and monotherapy for each study. **Supplementary Table 3.** Number of outcomes comparing recovery from DIC for combination therapy and monotherapy for each study. **Supplementary Figure 1.** Forest plot of random-effect analysis comparing mortality rates for combination therapy and monotherapy (AT or rTM) for septic DIC. A: Combination vs monotherapy (AT only). B: Combination vs monotherapy (rTM only). C: Combination vs monotherapy (AT only). D: Combination vs monotherapy (rTM only). E: Combination vs non-anticoagulant therapy. A, B and E: integrated hazard ratio results. C and D: integrated odds ratio results. Disseminated intravascular coagulation: DIC.

## Data Availability

The data and materials used in this meta-analysis are included in the references.

## Abbreviations

APC: Activated protein C
AT: Antithrombin
CI: Confidence interval
DIC: Disseminated intravascular coagulation
DPC: Diagnosis Procedure Combination
HR: Hazard ratio
ICHU-SHI: Igaku-Chuo Zasshi
ICU: Intensive care unit
OR: Odds ratio
RCT: Randomized controlled trial
rTM: Thrombomodulin
