# Supplementary material for: Effects of combination therapy of antithrombin and thrombomodulin for sepsis-associated disseminated intravascular coagulation: a systematic review and meta-analysis
Source: Thromb J. 2024 Jan 15;22:10. doi: 10.1186/s12959-023-00579-z (PMC10788990; doi:10.1186/s12959-023-00579-z)
Supplement: Supplementary file 1 — Additional file 1: Supplementary Table 1. Description of data: Specific details regarding the search strategies and results. Supplementary Table 2. Description of data: Number of outcomes comparing mortality rates for combination therapy and monotherapy for each study. Supplementary Table 3. Number of outcomes comparing recovery from DIC for combination therapy and monotherapy for each study. Supplementary Figure 1. Forest plot of random-effect analysis comparing mortality rates for combination therapy and monotherapy (AT or rTM) for septic DIC. A: Combination vs monotherapy (AT only). B: Combination vs monotherapy (rTM only). C: Combination vs monotherapy (AT only). D: Combination vs monotherapy (rTM only). E: Combination vs non-anticoagulant therapy. A, B and E: integrated hazard ratio results. C and D: integrated odds ratio results. Disseminated intravascular coagulation: DIC. [file 12959_2023_579_MOESM1_ESM.docx]

Supplement Table 1

PubMed search strategy（ Search date: January 14, 2023. ）

| #1 | Sepsis [Mesh] OR "Shock, Septic" [Mesh] OR "Systemic Inflammatory Response Syndrome" [Mesh] OR "Multiple Organ Failure" [Mesh] OR "Critical Illness" [Mesh] OR "Critical Care" [Mesh] OR "Intensive Care Units" [Mesh] OR "Purpura Fulminans"[Mesh] OR Thrombophilia [Mesh] OR Toxemia [Mesh] OR "Disseminated Intravascular Coagulation" [Mesh] OR Thrombocytopenia [Mesh] OR "Blood Coagulation Disorders" [Mesh] OR fibrinolysis [Mesh] OR infections [Mesh] OR bacteremia [Mesh] OR sepsis [tiab] OR (septic [tiab] AND shock [tiab]) OR "systemic inflammatory response syndrome" [tiab] OR SIRS [tiab] OR "organ dysfunction" [tiab] OR "organ failure" [tiab] OR MOF [tiab] OR "critically ill" [tiab] OR "critical illness" [tiab] OR "critical care" [tiab] OR "intensive care" [tiab] OR "Purpura Fulminans"[tiab] OR Thrombophilia [tiab] OR Toxemia [tiab] OR stressed [tiab] OR "Disseminated Intravascular Coagulation"[tiab] OR DIC [tiab] OR thrombocytopeni* [tiab] OR coagulopath* [tiab] OR (Coagulation [tiab] AND Disorder* [tiab]) OR fibrinoly* [tiab] OR hypercoagulab* [tiab] OR hypocoagulab* [tiab] OR hyperfibrinoly* [tiab] OR hypofibrinoly* [tiab] OR infection* [tiab] OR bacter* [tiab] | 4,827,333 |
| --- | --- | --- |
| #2 | Antithrombins [Mesh] OR "Antithrombin Proteins" [Mesh] OR "Antithrombin III" [Mesh] OR Antithrombin [tiab] OR "Antithrombin III"[tiab] OR "AT III" [tiab] | 30,874 |
| #3 | Thrombomodulin [Mesh] OR "recombinant human soluble thrombomodulin" [tiab] OR "recombinant thrombomodulin"[tiab] OR ART-123 [tiab] OR ART123 [tiab] OR thrombomodulin [tiab] OR "thrombomodulin alpha"[tiab] OR rTM [tiab] OR rhTM [tiab] OR recomodulin [tiab] | 5929 |
| #4 | animals [mh] NOT humans [mh] | 5,081,445 |
| #5 | #1 AND #2 AND #3 NOT #4 | 504 |

SCOPUS search strategy（ Search date: January 14, 2023. ）

| #1 | INDEXTERMS(Sepsis) OR INDEXTERMS("Shock, Septic") OR INDEXTERMS("Systemic Inflammatory Response Syndrome") OR INDEXTERMS("Multiple Organ Failure") OR INDEXTERMS("Critical Illness") OR INDEXTERMS("Critical Care") OR INDEXTERMS("Intensive Care Units") OR INDEXTERMS("Purpura Fulminans") OR INDEXTERMS(Thrombophilia) OR INDEXTERMS(Toxemia) OR INDEXTERMS("Disseminated Intravascular Coagulation") OR INDEXTERMS(Thrombocytopenia) OR INDEXTERMS("Blood Coagulation Disorders") OR INDEXTERMS(fibrinolysis) OR INDEXTERMS(infections) OR INDEXTERMS(bacteremia) OR TITLE-ABS(sepsis) OR (TITLE-ABS(septic) AND TITLE-ABS(shock)) OR TITLE-ABS("systemic inflammatory response syndrome") OR TITLE-ABS(SIRS) OR TITLE-ABS("organ dysfunction") OR TITLE-ABS("organ failure") OR TITLE-ABS(MOF) OR TITLE-ABS("critically ill") OR TITLE-ABS("critical illness") OR TITLE-ABS("critical care") OR TITLE-ABS("intensive care") OR TITLE-ABS("Purpura Fulminans") OR TITLE-ABS(Thrombophilia) OR TITLE-ABS(Toxemia) OR TITLE-ABS(stressed) OR TITLE-ABS("Disseminated Intravascular Coagulation") OR TITLE-ABS(DIC) OR TITLE-ABS(thrombocytopeni*) OR TITLE-ABS(coagulopath*) OR (TITLE-ABS(Coagulation) AND TITLE-ABS(Disorder*)) OR TITLE-ABS(fibrinoly*) OR TITLE-ABS(hypercoagulab*) OR TITLE-ABS(hypocoagulab*) OR TITLE-ABS(hyperfibrinoly*) OR TITLE-ABS(hypofibrinoly*) OR TITLE-ABS(infection*) OR TITLE-ABS(bacter*) | 4,990,073 |
| --- | --- | --- |
| #2 | INDEXTERMS(Antithrombins) OR INDEXTERMS("Antithrombin Proteins") OR INDEXTERMS("Antithrombin III") OR TITLE-ABS(Antithrombin) OR TITLE-ABS("Antithrombin III") OR TITLE-ABS("AT III") | 28,583 |
| #3 | INDEXTERMS(Thrombomodulin) OR TITLE-ABS("recombinant human soluble thrombomodulin") OR TITLE-ABS("recombinant thrombomodulin") OR TITLE-ABS(ART-123) OR TITLE-ABS(ART123) OR TITLE-ABS(thrombomodulin) OR TITLE-ABS("thrombomodulin alpha") OR TITLE-ABS(rTM) OR TITLE-ABS(rhTM) OR TITLE-ABS(recomodulin) | 15,381 |
| #4 | INDEXTERMS(animals) NOT INDEXTERMS(humans) | 986,144 |
| #5 | #1 AND #2 AND #3 AND NOT #4 | 691 |

CENTRAL search strategy（ Search date: January 14, 2023. ）

| #1 | MeSH descriptor: [Sepsis] explode all trees | 5021 |
| --- | --- | --- |
| #2 | MeSH descriptor: [Shock, Septic] explode all trees | 1097 |
| #3 | MeSH descriptor: [Systemic Inflammatory Response Syndrome] explode all trees | 5428 |
| #4 | MeSH descriptor: [Multiple Organ Failure] explode all trees | 443 |
| #5 | MeSH descriptor: [Critical Illness] explode all trees | 2694 |
| #6 | MeSH descriptor: [Critical Care] explode all trees | 2245 |
| #7 | MeSH descriptor: [Intensive Care Units] explode all trees | 4158 |
| #8 | MeSH descriptor: [Thrombophilia] explode all trees | 376 |
| #9 | MeSH descriptor: [Toxemia] explode all trees | 636 |
| #10 | MeSH descriptor: [Disseminated Intravascular Coagulation] explode all trees | 114 |
| #11 | MeSH descriptor: [Thrombocytopenia] explode all trees | 1406 |
| #12 | MeSH descriptor: [Blood Coagulation Disorders] explode all trees | 1881 |
| #13 | MeSH descriptor: [Fibrinolysis] explode all trees | 1010 |
| #14 | MeSH descriptor: [Infections] explode all trees | 83577 |
| #15 | MeSH descriptor: [Bacteremia] explode all trees | 1050 |
| #16 | (Sepsis):ti,ab,kw | 12750 |
| #17 | (“Septic Shock”):ti,ab,kw | 3473 |
| #18 | (“Systemic Inflammatory Response Syndrome”):ti,ab,kw | 1215 |
| #19 | (SIRS):ti,ab,kw | 846 |
| #20 | (“Organ dysfunction”):ti,ab,kw | 2025 |
| #21 | (“Organ Failure”):ti,ab,kw | 3963 |
| #22 | (MOF):ti,ab,kw | 193 |
| #23 | (“Critically Ill”):ti,ab,kw | 8058 |
| #24 | (“Critical Illness”):ti,ab,kw | 4162 |
| #25 | (“Critical Care”):ti,ab,kw | 4562 |
| #26 | (“Intensive Care”):ti,ab,kw | 28122 |
| #27 | (“Purpura Fulminans”):ti,ab,kw | 16 |
| #28 | (Thrombophilia):ti,ab,kw | 495 |
| #29 | (Toxemia):ti,ab,kw | 457 |
| #30 | (Stressed):ti,ab,kw | 1305 |
| #31 | (“Disseminated Intravascular Coagulation”):ti,ab,kw | 384 |
| #32 | (DIC):ti,ab,kw | 385 |
| #33 | (Thrombocytopeni*):ti,ab,kw | 11517 |
| #34 | (Coagulopath*):ti,ab,kw | 1689 |
| #35 | (“Coagulation Disorders”):ti,ab,kw | 822 |
| #36 | (Fibrinoly*):ti,ab,kw | 6946 |
| #37 | (Hypercoagulab*):ti,ab,kw | 699 |
| #38 | (Hypocoagulab*):ti,ab,kw | 45 |
| #39 | (Hyperfibrinoly*):ti,ab,kw | 90 |
| #40 | (Hypofibrinoly*):ti,ab,kw | 39 |
| #41 | (Infection*):ti,ab,kw | 133628 |
| #42 | (Bacter*):ti,ab,kw | 48566 |
| #43 | #1 OR #2 OR #3 OR #4 OR #5 OR #6 OR #7 OR #8 OR #9 OR #10 OR #11 OR #12 OR #13 OR #14 OR #15 OR #16 OR #17 OR #18 OR #19 OR #20 OR #21 OR #22 OR #23 OR #24 OR #25 OR #26 OR #27 OR #28 OR #29 OR #30 OR #31 OR #32 OR #33 OR #34 OR #35 OR #36 OR #37 OR #38 OR #39 OR #40 OR #41 OR #42 | 238213 |
| #44 | MeSH descriptor: [Antithrombins] explode all trees | 1009 |
| #45 | MeSH descriptor: [Antithrombin Proteins] explode all trees | 848 |
| #46 | MeSH descriptor: [Antithrombin III] explode all trees | 497 |
| #47 | (Antithrombin):ti,ab,kw | 1927 |
| #48 | (Antithrombin III):ti,ab,kw | 1146 |
| #49 | (AT III):ti,ab,kw | 103333 |
| #50 | #44 OR #45 OR #46 OR #47 OR #48 OR #49 | 105143 |
| #51 | MeSH descriptor: [Thrombomodulin] explode all trees | 110 |
| #52 | (Thrombomodulin):ti,ab,kw | 398 |
| #53 | (ART-123):ti,ab,kw | 41 |
| #54 | (ART123):ti,ab,kw | 1 |
| #55 | (rTM):ti,ab,kw | 93 |
| #56 | (rhTM):ti,ab,kw | 20 |
| #57 | (hrTM):ti,ab,kw | 6 |
| #58 | (Recomodulin):ti,ab,kw | 1 |
| #59 | #49 OR #50 OR #51 OR #52 OR #53 OR #54 OR #55 OR #56 | 494 |
| #60 | MeSH descriptor: [Animals] explode all trees | 658869 |
| #61 | MeSH descriptor: [Humans] explode all trees | 658856 |
| #62 | (Animals):ti,ab,kw | 16016 |
| #63 | (Humans):ti,ab,kw | 671525 |
| #64 | (#58 OR #60) NOT (#59 OR #61) | 2259 |
| #65 | #43 AND #48 AND 57 NOT #62 | 66 |

Supplement Table 2

|  | Combination therapy | | Mono therapy | |
| --- | --- | --- | --- | --- |
|  | Death | Total | Death | Total |
| Morita | 2 | 40 | 0 | 16 |
| Takehara | 2 | 8 | 2 | 5 |
| Sakurai | 3 | 20 | 10 | 40 |
| Hosomi | 0 | 10 | 0 | 10 |

Supplement Table 3

|  | Combination therapy | | Mono therapy | |
| --- | --- | --- | --- | --- |
|  | Recovery | Total | Recovery | Total |
| Morita | 36 | 40 | 16 | 16 |
| Takehara | 7 | 8 | 3 | 5 |
| Sakurai | 17 | 20 | 30 | 40 |

Supplement Figure 1-A: Combination vs mono therapy (AT only)

Supplement Figure 1-B: Combination vs mono therapy (rTM only)

Supplement Figure 1-C: Combination vs mono therapy (AT only)

Supplement Figure 1-D: Combination vs mono therapy (rTM only)

Supplement Figure 1-E: Combination vs Non-anticoagulant therapy
